# “Be rational!” Epistemic aims and socio-cognitive tension in argumentation about dietary choices

**DOI:** 10.3389/fpsyg.2022.933062

**Published:** 2022-10-11

**Authors:** Pablo Brocos, María Pilar Jiménez-Aleixandre, Michael J. Baker

**Affiliations:** ^1^Departamento de Didácticas Aplicadas, Universidade de Santiago de Compostela, Santiago de Compostela, Spain; ^2^Centre National de la Recherche Scientifique, UMR 9217 i3, Télécom Paris, Paris, France

**Keywords:** epistemic cognition, argumentation, classroom discourse, socio-cognitive conflict, collaborative learning

## Abstract

Argumentation is a social practice that can lead to epistemic outcomes, that is, to the construction of knowledge. Recent research in collaborative learning has pointed out the significance of affective and motivational aspects, as well as the influence of socio-relational concerns, which have been found to frequently take priority over epistemic ones. Our research objective is to investigate how the epistemic and socio-relational dimensions of students' argumentative interactions are intertwined. We apply discourse analysis to examine the interactions in a small group of four 11th-graders evaluating the nutritional acceptability of omnivorous and vegetarian diets. The epistemic dimension is analyzed in terms of the aims pursued by the participants and the epistemic outcomes achieved. The socio-relational dimension is analyzed in terms of fluctuations of interpersonal tensions and their relaxations. The results show a convergence of participants' epistemic aims and the epistemic statuses of the options. Most of the epistemic outcomes are produced in sequences in which socio-cognitive tension arises and then relaxes. Enduring high socio-cognitive tension and overcoming conflict seem to have encouraged the adoption of epistemic aims. Moreover, our findings suggest that driven by epistemic aims in high socio-cognitive tensed contexts, students can refine the conditions by which they engage in argumentation. These results call for further investigating on what constitutes an appropriate or productive level of interpersonal tension for learning. Educational implications are related to the design of argumentative learning environments promoting epistemic aims and outcomes through the encouragement of suitable socio-cognitive climates leading to them.

## Introduction

Research on epistemic cognition (EC), that is, on knowledge about how knowledge is constructed and justified, has developed in recent years (Chinn et al., 2014; Greene et al., 2016a), showing that it is situation-specific and pointing out the need for further examination of patterns across specific practices and contexts. There has been a shift from conceptualizing EC as essentially individual toward a consideration of its social nature (Goldman, 1999; Ludvigsen, 2009; Chinn and Rinehart, 2016). However, its social aspects, such as how individual practices aggregate at the level of groups, are still understudied (Asterhan, 2013; Chinn et al., 2014; Clément, 2016).

Argumentation has a range of meanings, and several types of argumentative dialogue have been described in the literature (Walton, 1990). In relation to EC, argumentation can be defined as an epistemic and social practice in which interlocutors attempt to modify the intersubjectively agreed degree of acceptability, or, more generally, the *epistemic status* of proposals (claims, views, ideas, or solutions) through joint engagement in reasoning (Asterhan, 2013). Being an inherently social practice taking place in the interplay between people, argumentation is a suitable context for studying the social aspects of EC. Given the appropriate conditions, argumentation can lead to *epistemic ends, outcomes*, or *achievements*, that is, to the construction of new knowledge. This newly acquired understanding may be achieved through the modification of the epistemic statuses of the ideas in discussion (that is, the intelligibility, plausibility, or acceptability of those ideas, from the point of view of each participant) when interlocutors publicly present their ideas and resolve discrepancies. Indeed, research has shown that argumentation can be an effective way of learning and refining understanding (Nussbaum and Sinatra, 2003; Clark and Sampson, 2008; Felton et al., 2009; Yeh and She, 2010). However, there is a need for further research on how argumentation produces specific changes in the consideration of ideas in their epistemic statuses. Research shows that it can do so, although not always (Asterhan and Schwarz, 2007; Goldman et al., 2016), and clear changes in position (e.g., from being in favor to being against a proposal) are, in fact, a rare finding, especially in socio-scientific debates involving systems of values (Simonneaux, 2001; Yu and Yore, 2013). Another kind of knowledge, besides the one specifically related to the epistemic status of the ideas in discussion, may be constructed as a result of argumentation: knowledge about the knowledge-building processes themselves. While it has been shown that engagement in argumentation supports the development of epistemic understanding (Kuhn et al., 2013; Iordanou and Constantinou, 2015), that is, meta-level understanding of the construction of knowledge, further research is required to address the complexity of the interconnections between the emergence of this kind of epistemic achievements and the engagement in argumentation (Iordanou et al., 2016).

Two areas of research are relevant for understanding why argumentative interactions do or do not consistently produce epistemic achievements. Both involve the acknowledgment of the “hot” nature of learning and cognition (Bendixen and Rule, 2004; Sinatra, 2005) encompassing affective and motivational components (Chen and Barger, 2016; Brocos and Jiménez-Aleixandre, 2021). The first area concerns the goals and motivation behind EC, exploring the question of why individuals would want to engage in thinking about knowledge and knowing. In this regard, the interest in the AIR model of EC developed by Chinn et al. (2011, 2014) has been emphasized (Chen and Barger, 2016). This model, described below, incorporates as one of its essential components *epistemic aims*, which are goals that individuals may adopt in relation to knowledge. Thus, this model incorporates motivational constructs into EC (Chen and Barger, 2016). As the relevance of epistemic aims in knowledge construction has been recognized, they are likely to play an important role in determining the extent to which epistemic outcomes are achieved in argumentative exchanges. Since collaborative engagement is not the mere sum of the individual's motivation levels, being influenced by group dynamics (Mullins et al., 2013), epistemic aims are arguably subjected to the particularities of how the social interaction unfolds, and as such, they should be examined.

The second research area of interest to our research purposes concerns the social aspects of argumentation. While much attention has been paid to the cognitive and epistemic dimensions of argumentation, the socio-relational dynamics on which these dimensions are dependent have been largely understudied (Andriessen et al., 2011; Asterhan, 2013). While engaged in argumentation, participants are not only concerned with epistemic matters related to the issue under discussion, but also with aspects related to social belongingness, interpersonal relations, and social perceptions (Hijzen et al., 2007; Asterhan, 2013). It has been pointed out that these socio-relational aspects often take priority over epistemic ones (Andriessen et al., 2011; Asterhan, 2013; Isohätälä et al., 2018), demonstrating that emotional tension frames the construction of arguments (Brocos and Jiménez-Aleixandre, 2021). Research has yet to further clarify how engaging in productive argumentation while regulating socio-emotional processes occurs and intertwines in students' interactions (Baker et al., 2007). In this regard, examining the patterns of *socio-cognitive tension* and its relaxation in argumentative interactions has been proposed as a potentially insightful approach to better understanding whether and how learning occurs in argumentative exchanges (Andriessen et al., 2011). Thus, we incorporate the analysis of socio-cognitive tensions in our study.

In sum, our research objective is to investigate how the epistemic and socio-relational dimensions of students' argumentative interactions are intertwined. In particular:

To examine in which ways enduring socio-cognitive tension and overcoming conflict in group argumentation encourages or inhibits the adoption of epistemic aims and the achievement of epistemic outcomes.

The epistemic dimension is analyzed in terms of the epistemic or non-epistemic aims pursued by the participants, and in terms of the epistemic outcomes achieved. The socio-relational dimension is analyzed in terms of fluctuations of interpersonal tensions and their relaxations.

We pursue this objective through a case study, examining the social interactions of a group of four 11th-grade students participating in a socio-scientific argumentation task involving the evaluation of dietary options. This group was selected since an analysis of their argumentative exchanges suggested that they achieved sophisticated epistemic outcomes, both in terms of deepening the knowledge about the matter at stake, as well as in terms of meta-level understanding, as they engaged in explicit regulation of the argumentative and decision-making processes. We examined the evolution of the epistemic status of their proposals relating to dietary choices (vegetarian, omnivorous) throughout the debate, in relation to the epistemic aims of the participants, the patterns of socio-cognitive tension, and the regulation of epistemic processes. We outlined the possible interactions among these dimensions, delving into the specific ways in which these may be interconnected. We did so by analyzing excerpts of the participants' discourse, which marked the progression of the discussion toward the outcome of the task. In so doing, we intended to shed some light on how the interrelation of participants' epistemic aims and the particularities of the socio-cognitive climate may interrelate and influence the epistemic outcomes achieved in argumentative exchanges.

## Theoretical framework

The framework is drawn from the literature on epistemic cognition and research on socio-relational aspects of argumentation and collaborative learning.

### Argumentation as a social epistemic practice

EC is an interdisciplinary research area that addresses how people acquire, understand, justify, change, and use knowledge, having its roots in psychology, sociology of science, and philosophy (Greene et al., 2016b). EC has also been presented as a theory of personal epistemology (Hofer and Pintrich, 2002), epistemological resources (Hammer and Elby, 2002), or of the nature of science (Osborne et al., 2003). Perspectives on how knowledge is produced are shifting from an individual focus toward a social one (Ludvigsen, 2009; Chinn and Rinehart, 2016; Clément, 2016). Simultaneously, the field has expanded from personally held beliefs about knowledge to a broad spectrum of cognitive processes (Chen and Barger, 2016). In recent years, new models for EC have been developed, aiming at a better understanding of how beliefs change. These models are potentially helpful for researching and explaining the role of social, affective, and motivational aspects of EC that have been traditionally neglected (Asterhan, 2013). Chen and Barger (2016) model of epistemic change comprises three components: epistemic doubt (being skeptical of one's beliefs), epistemic volition (related to motivational aspects), and resolution strategies such as reflection, social support, and social interaction. The latter two components deconstruct the roles of motivational and social aspects of EC, where, as these authors emphasized in earlier studies, the role of peers and emotions in epistemic change needs to be further elucidated.

More recently, Chinn et al. (2014) developed the AIR model of EC, which includes three components: *Aims*, epistemic goals or objectives that individuals set to pursue epistemic ends; *Ideals*, standards used to evaluate whether epistemic ends have been achieved; and *Reliable epistemic processes*, procedures, and strategies to achieve epistemic ends. The notion of epistemic ends, also termed epistemic products, outcomes, or achievements, refers to the new knowledge or understanding that is being constructed in each situation. According to this model, when processing information, individuals may adopt epistemic aims; for instance, they may set goals for developing a representation of how the world is (Chinn and Rinehart, 2016). Conversely, and perhaps simultaneously, they might adopt non-epistemic aims that are not specifically related to knowledge and may be diverse in nature, for instance, concerning personal pleasure or self-image. Chen and Barger (2016) illustrated the differences between both kinds of aims with the following example: students can be oriented to understand the biases of the author of a text to better understand the complexity of the issue (an epistemic aim) or to be esteemed by their peers for finding an interesting insight (a non-epistemic aim). We can map the other two elements of the AIR model onto the same example: students could use certain epistemic ideals as criteria for deciding what constitutes a bias, and enact specific epistemic processes, such as systematically consulting a range of sources to acquire information about the author and hence gain a better understanding of the author's biases.

Epistemic and non-epistemic aims interact, and it has been argued that people are often driven by a mix of the two (Kawasaki et al., 2014), given that, in group work, they must simultaneously manage problem-solving and social interaction. The notion of epistemic aims involves both motivational and social aspects of EC. Thus, the AIR model incorporates goal-orientation constructs traditionally studied in the motivation literature (Maehr and Zusho, 2009) into EC, further expanding them by including not only features related to what motivates people, but also what people value when dealing with epistemic matters (Chen and Barger, 2016). The AIR model highlights that EC is social and contextualized and that it is centered on practices rather than formal beliefs (Chinn and Rinehart, 2016). In this study, we aim to contribute to research on EC by studying the epistemic practice of argumentation in a socio-scientific context, exploring how the epistemic and non-epistemic aims of participants might be related to socio-relational aspects and the epistemic outcomes are potentially achieved because of argumentative exchanges.

Argumentation in science education is conceptualized as an epistemic practice that involves the evaluation of knowledge. It has the potential to broaden, deepen, and refine understanding, as it may foster justification, negotiation of meaning, and opinion (epistemic status) change (Baker, 2009). We should clarify that we use the term *epistemic status* as it is understood within EC, referring to the status of ideas from a participant's point of view, and it should not be mistaken with the use of the same term in the conversation analysis discipline, in which it is generally utilized to characterize the relative position of speakers in a gradient of knowledge about the domain in discussion (Heritage, 2012; Lindwall et al., 2016). In argumentative situations, proposals will have different epistemic statuses from the participants' points of view. The aim of the argumentative interaction is to try to make epistemic statuses evolve so that agreement is reached on what should be mutually accepted (Baker, 2002), so the epistemic status of the ideas in discussion from each participant's point of view is better aligned. We argue that the term *epistemic status* has a range of meanings in diverse argumentative contexts. In developing explanations, it refers to the plausibility and explanatory power of alternative models, and, in decision-making, to the degree of *acceptability* of options. It is worth mentioning that, as Kolstø (2005) argues, decision-making is not solely based on knowledge, but a result of the interaction between knowledge and values, the latter being necessary for assessing the desirability of the different potential consequences of alternative decisions. In these contexts, argumentative interactions have the potential to modify the epistemic statuses associated with the alternative options (that is, their acceptability for each participant), in terms of their consistency with other conceptions and values (individually or socially accepted), their consistency with evidence, or their potential to successfully address several dimensions of the dilemma and achieve something that is considered of value. We further explore the differences between the shifting of epistemic statuses in the context of scientific explanations and socio-scientific decision-making in another study (Jiménez-Aleixandre and Brocos, 2018).

Thus, epistemic statuses may be modified as an outcome of argumentation, so the argumentative interactions can be characterized as discursive moves aimed at triggering acceptability changes, or as attempts to decide on alternative solutions by transforming attitudes toward them (Baker, 2009). When students express information and reasoning relating to a problem, they potentially change the degrees of acceptability of the options being discussed and they presumably construct new knowledge (Baker, 2009). Viewed thus, not only is the interactive epistemic process (argumentation) social in nature, but the epistemic ends achieved by argumentative means (understanding) are social as well.

Argumentation may be a reliable process for achieving epistemic ends. However, as Chinn et al. (2014) point out, its reliability depends on certain conditions. We argue that these conditions are closely related to what Baker (2009) has termed the argumentative rules of the dialogue game. Some of these rules are *logical*, such as the requirement for coherence (e.g., invalidating an argumentative position if it has incurred a contradiction), while others relate to the *collaborative* nature of argumentation (e.g., dismissing a party who argues in circles, allowing no evolution of the debate, or the obligation of defending one's position when it is criticized). In the pragma-dialectical perspective, the “ten commandments” for a critical discussion (van Eemeren and Grootendorst, 1992) can be considered as a set of rules for engaging in argumentation in a reliable way. Chinn et al. (2011) point out that people may have ideas about the conditions, generally tacit (Baker, 2009), that must be met in small-group procedures to reliably produce epistemic outcomes. By engaging in practices such as argumentation, individuals may develop these ideas, and hence a better understanding of the procedures themselves. So, by practicing argumentation, individuals may achieve not only epistemic ends about what is being argued but also about the argumentation process itself. Indeed, it has been shown that engagement in argumentation supports the development of meta-level knowledge (Kuhn et al., 2013; Iordanou and Constantinou, 2015), and research on students' metatalk (talk about the discourse, distinguished from talk about the topic) sheds some light on the rules that govern argumentative exchanges, showing that, over sustained periods, students' discourses become more explicit regarding norms (Kuhn et al., 2008, 2013). Further research is required to address the complexity of the interconnections between the development of meta-level knowledge and engagement in argumentation (Iordanou et al., 2016). In our study, we address how the development of this kind of knowledge might relate to socio-relational dynamics.

### The socio-relational dimension of argumentation

It should be noted that co-construction of knowledge in argumentation does not necessarily emerge from an initial disagreement, and all that is required is a diversity of proposals and epistemic statuses ascribed to them. However, research shows that conceptual gains are primarily predicted by the presence of critical aspects of argumentative discourse, such as contradiction or rebuttals, and less so by purely consensual reasoning moves (Asterhan and Schwarz, 2007, 2009a; Howe, 2009). Asterhan (2013) argues that for argumentation to be conducive to learning, it should be both critical and constructive, coining the notion of co-constructive critical argumentation (Asterhan and Schwarz, 2009b), related to “deliberative argumentation” (Asterhan and Schwarz, 2016), “exploratory talk” (Mercer, 1996), or “collaborative argumentation” (Nussbaum, 2008). Asterhan (2013) points out that co-constructive critical argumentation includes features such as (a) willingness to listen and critically examine the different ideas and alternatives proposed; (b) willingness to make concessions; (c) competition between ideas, rather than individuals; and (d) a collaborative and respectful atmosphere. These features, we argue, are largely dependent on the balance between epistemic and non-epistemic aims of interlocutors, their motivations, goals, and willingness. Indeed, Asterhan (2013), discussing why this kind of productive argumentation might be so difficult to elicit in educational contexts, points to the relevance of conflicts between different goals, indicating that concerns about interpersonal relations and social perceptions may cause students to primarily focus on the social dimension on the conflict, rather than on the epistemic one. We must consider, then, the inherent socio-relational aspects of argumentative interactions.

Labov and Fanshel (1977) argue that the kind of conversational actions with the greatest social impact is those in connection with the status of participants and their changing social relationships. When argumentation arises from disagreement, it is potentially a face-threatening activity (Grimshaw, 1990) where criticism of a person's views can carry with it an element of indirect criticism of the person proposing them. However, according to Muntigl and Turnbull (1998), arguing does not necessarily damage social relations, as it can also strengthen group bonds. They propose that, as facework concerns potentially both positive and negative social relations, it may play an important role in how argumentative exchanges are conducted.

Productive interaction in argumentation requires a balance between engaging in high-level cognitive processes, which are potentially critical and confrontational while sustaining favorable socio-emotional processes (Isohätälä et al., 2018), as students require a workable relationship with their partners (Andriessen et al., 2011). The greater the difference in interlocutors' knowledge and intentions, the greater the socio-cognitive tension in the working relationship, but also the more potential mutual gain (Andriessen et al., 2011). Avoiding confrontation and tension altogether does not provide grounds for high-level critical discussion which may imply missing learning opportunities (Isohätälä et al., 2018). Thus, socio-relational concerns may divert students' attention away from the epistemic dimensions, resulting in argumentative discourse that can be too critical on an interpersonal level, and uncooperative, or else too consensual, and hence devoid of the criticism needed for co-construction of knowledge (Asterhan, 2013). If arguers can deal with the interpersonal aspect, they may develop their ideas whereby tuning at the epistemic level may be related to tuning at the socio-cognitive level (Andriessen et al., 2011). It has been found in certain cases that socio-relational aspects are prioritized over epistemic concerns (Andriessen et al., 2011; Asterhan, 2013), but further research is needed to understand how students deal with socio-cognitive tension, and for uncovering how and under what conditions this tension may allow or facilitate the construction of knowledge.

## Methods

### Research design

This study adopts a qualitative methods approach, seeking to analyze educational cases through expressions and actions in their local contexts (Denzin and Lincoln, 2013). We present a case study examining interactions in a group of four 11th-graders. Data collection included written products and video recordings, through immersion of the first author in the classroom during 22 sessions of the project. For our study, the data corpus comprises one small group's written evaluation and video recordings of two sessions. This micro-analytic approach is appropriate given the need for fine-grained analysis for exploring EC in the context of social interaction *in situ* (Bendixen and Rule, 2004; Chen and Barger, 2016; Iordanou et al., 2016), in which measures need to be context, task, and even case-specific (Bendixen and Rule, 2004; Chen and Barger, 2016; Isohätälä et al., 2018).

### Participants

Participants were drawn from an interdisciplinary project on food choices, carried out with the complete cohort of 11th-grade students in a high school, aged 16–18 years, during a school year. They were 35 students (22 girls and 13 boys), from sciences and humanities, divided into eight small groups. The case study examined interactions in one group of four 11th-graders (two girls and two boys), participating in a task as part of the final phase of the project. The participants, identified by pseudonyms, were already familiar with each other. This particular small group was selected for micro-analysis based on the following criteria: (a) diversity and changes in the group members' epistemic and non-epistemic aims; (b) fluctuating socio-cognitive environment in terms of tension-relaxation; and (c) abundance of epistemic outcomes achieved. These outcomes included explicit modifications of the scoring of the options at stake (different dietary choices), argumentative broadening and deepening of the notions discussed, as well as regulation of the epistemic processes of argumentation and decision-making. It should be noted that this group was not meant to be representative, particularly due to the higher density and diversity of the epistemic outcomes achieved in their interactions, which was mainly noticeable in the sequence of 161 turns selected for our in-depth analysis. We were primarily interested in unraveling what specifically happened in this group that led to sophisticated epistemic outcomes, and in doing so, we hoped to refine our understanding of the conditions under which these outcomes were achieved in argumentative interactions. Following the aforementioned criteria for group selection, we believed that the analysis of the selected group held the potential for answering our research questions and allowing us to explore whether and how epistemic outcomes that were achieved in argumentative exchanges might relate to the interlocutors' epistemic aims and the socio-cognitive climate.

### Context: Project on healthy and sustainable food choices

The project on healthy and sustainable food choices was carried out in a high school in a small town where the main activity is agriculture and livestock breeding, including a milk factory. Its aim was two-fold: (a) to promote students' development of the practice of argumentation, and (b) to encourage critical and informed decision-making on dietary options (vegetarian, omnivorous) based on five criteria including nutritional, environmental, economic, ethnic, and cultural/personal. The project design aimed at promoting the understanding of the environmental impact of different diets, which has been recently researched and emphasized in the sustainability literature (Stehfest et al., 2009; Tilman and Clark, 2014). The results of the participants' consideration of the environmental impact of diets and their use of environmental data are addressed in another paper (Brocos and Jiménez-Aleixandre, 2020a). The project consisted of three phases: (1) practicing argumentation and identifying criteria for an optimal diet, (2) searching for information and discussing the criteria, and (3) undertaking the argumentation task on diets carried out in three 50-minute sessions, which is the focus of this paper. This teaching sequence is further detailed in Brocos and Jiménez-Aleixandre (2020b).

The task was designed according to the optimization strategy (Papadouris, 2012), which provides a framework for the evaluation of options. The students were asked to use the information gathered during the second phase of the project pertaining to the five criteria to discuss within the group and jointly evaluate omnivorous and vegetarian diets. They had to score (0–10) on each criterion and provide a written justification for each score. This paper is focused on the discussion around the nutrition criteria. The group was engaged in this task in the first session for 50 mins and an additional 20 mins in the second session. The turns cited from the second session are preceded by “S2-”.

### Data analysis

We employed the microgenetic approach, which involves a close examination of the participants' discursive interchanges during practice, focusing on moment-to-moment interaction. Researchers have recommended this method to obtain a comprehensive understanding of epistemic development (Iordanou and Constantinou, 2015; Iordanou et al., 2016) as it can provide insight into the processes of change (Sandoval, 2014). Analysis of the categories was constructed iteratively, analyzing the data in several cycles and in interaction with the literature.

First, the sessions were transcribed. The coding was conducted using the transcripts in the two languages in which the discourse was originally produced, Spanish and Galician, and it was mainly carried out by the first two authors, who are bilingual and hence fully proficient in both languages. The third author worked with English translations. The unit of analysis was the speech turn. Turns were grouped into episodes, defined as one or several turns of speech related to the same topic or action (Gee, 2014). Transcriptions and written productions were analyzed through prolonged immersion in the data. Initial repertoires of categories were elaborated, drawing from the literature, and independently assigning a tentative code to each unit. The codes were compared and the differences were resolved. Then the categories were refined. Using these revised categories, data were subjected to several cycles of analysis. Selected fragments translated to English are reproduced to illustrate the analysis.

In this manner, the first session was divided into 12 episodes. We focused on the analysis of episodes one to five from turns 1–161 as they corresponded to the sequences in which we found a higher density of epistemic outcomes, both in terms of changes in epistemic statuses, refinement of the conceptual notions discussed, and regulation of epistemic processes; these were obtained within a fluctuating socio-cognitive environment.

#### Analysis of epistemic outcomes

We analyzed three kinds of epistemic outcomes. Firstly, outcomes related to the *modification of the epistemic statuses* of the options at stake. As discussed, argumentation can be interpreted as a practice in which the participants attempt to modify the epistemic status of ideas in the discussion. In decision-making contexts, the ideas at stake are the specific options or decisions being considered. The epistemic statuses of those options correspond to their acceptability, that is, the degree to which they are considered adequate, viable, and desirable in the context of the specific issue being debated. The task demanded that participants score each criterion according to its adequacy, so the scores publicly manifested by each participant during the debate can be considered as an indicator of the acceptability of that option for that participant, i.e., the epistemic status of that option at that time for that participant. Thus, we examined the evolution of the epistemic statuses by tracking the scores publicly proposed and accepted throughout the discussion, as they indicated the *degrees of acceptability* of each option for the participants who proposed or accepted such scores at a given time in the debate.

Secondly, epistemic outcomes related to the constructed arguments and what has been termed *broadening and deepening of the space of debate* (Baker et al., 2007). These outcomes are constructed by producing an argument, or a counter-argument, or by discussing argumentative links, or the meaning of key notions that a given argument is built on discursive operations such as reformulation, conceptual dissociation, association, or elaboration. In our analysis, we represent the constructed arguments and the argumentative operations in a diagrammatic form.

Thirdly, we analyzed the epistemic outcomes related to the *regulation of the conditions* under which the argumentation and decision-making practices were carried out. In particular, we focused on how the *rules of debate* were established during the discussion and how the legitimacy of argumentative moves was regulated. We identified these rules when students pointed out that from their point of view a peer had violated the rule. The rules included those related to the requirement of logical consistency, the cooperative nature of argumentation, and the delimitation of the scope of the debate (i.e. what is or is not relevant to the debate).

#### Analysis of epistemic and non-epistemic aims

To access the epistemic aims adopted by the participants, which require inferring underlying intentions from their discursive moves, the data were examined by fine-grained discourse analysis in an interpretive process that requires elucidation within the sequence context, rather than consideration of separate, individual turns. From the theoretical grounds established in the AIR model developed by Chinn et al. (2011), and in interaction with the data analyzed, we built a non-comprehensive coding scheme (Table 1) for characterizing the participants' aims expressed through their interactions in the debate. We coded as epistemic the utterances signaling aims directed at cognitive representational goals. The non-epistemic aims category encapsulated aims of diverse nature unrelated to the construction of knowledge, including utterances that suggest pragmatic goals such as finishing the task as soon as possible or preserving a positive self-image. As we cannot directly access the participants' aims, we identify them by analyzing participants' engagement in certain epistemic performances revealed in their discourse. Table 1 summarizes the coding scheme for the analysis of the epistemic and non-epistemic aims, including a non-exhaustive list of performances indicative of them. It must be noted that the analyses of epistemic outcomes, epistemic aims, and socio-cognitive tension are not mutually exclusive. For instance, a specific utterance in which a participant regulates the rules of debate implies the achievement of epistemic outcomes related to the regulation of the argumentation practice, but it is also considered an epistemic performance, and hence, interpreted as indicative of epistemic aims. Alternatively, certain utterances signaling non-epistemic aims might as well be considered discursive moves increasing socio-cognitive tension. The analysis of the participants' aims was independently conducted by the first two authors, showing an agreement of 93% and a Cohen's kappa value of 0.88.

**Table 1:** Coding categories for epistemic and non-epistemic aims and students' performances indicative of them.

**Aims**	**Description**	**Examples of performances indicative of epistemic/non-epistemic aims**	**Instances from the students' discourse**
Epistemic aims	Considering relevant evidence	Manifesting disposition to consider additional evidence	*47 Elena: But no, look, for example, it says… where we can find it [zinc in food]*
	Achieving a properly justified claim	Providing justifications for the score proposed	*75 Elisa: I would give it a 7 […]It supplies everything, but […] it can lead to cardiovascular diseases*
	Interpreting the information accurately	Detecting errors in the data handout	*S2-158 Santiago: Here there is an incongruity!*
	Achieving collective understandings	Asking questions to understand a peer's reasoning or to clarify the meaning of a concept	*49 Elena: You mean like mixing lentils with rice and all that…?*
	Engaging in reliable epistemic processes	Encouraging a peer to follow the proper rules to reliably engage in epistemic processes	*114 Alfonso: Come on, man, speak right, I'm speaking right, dude*
Non-epistemic aims	Finishing the task as soon as possible	Restricting the amount or quality of the evidence considered	*35 Alfonso: What difference does it make… let's move on, let's not stop at that*
	Preserving a positive self-image	Preventing the modification of the epistemic status of preconceived ideas with personal implications	*58 Santiago: But no, look, each one of us gives a score, and then we do the average*
	Prioritizing lack of effort over the quality of the result	Making decisions or proposals based on how easy they are to be justified	*316 Santiago: Let's see, meh… explaining a 10 is easier. Put a 10*
	Achieving high scores in the subject	Expressing concerns about the relevance of the task for the subject scores	*196 Alfonso: This is taken into account for our scores. It is important*
	Enjoying oneself	Engaging in activities unrelated to the task such as making jokes or playing games	*S3-239 Elisa: You have white hair on the ear!*
Uncodifiable		Utterances that are not indicative of either epistemic or non-epistemic aims	*38 Elisa: Mine is here*

#### Analysis of socio-cognitive tension

Drawing from Andriessen et al. (2011, 2013), we examined socio-cognitive tension as arising from argumentation and disagreement-in-discourse. We analyzed the potential of a broad inventory of discursive moves to negotiate face and increase or decrease socio-cognitive tension, which is summarized in Table 2. This repertoire builds on the work of Andriessen et al. (2011), adapted to the particularities of our data, and ranged from metatalk utterances (Kuhn et al., 2013), such as meta-directive statements, to more traditional argumentative moves, like counterarguments or concessions, as well as discursive moves involving affection, such as the use of displayed emotions (Plantin, 2011), humor, or irony. These are used to estimate the evolution of the tension-relaxation pattern throughout the argumentative exchanges, which, at a given point in the debate, is quantified as the number of tensed utterances minus the number of relaxed responses. This quantification is a simplification and represents the general direction of tension increase or decrease. It should be noted that: (a) the socio-cognitive potential of certain utterances is highly context-dependent, for instance, humor can be used in either a playful manner, reducing tension, or in a hurtful manner, increasing it (b) certain utterances might include elements that may simultaneously increase and relax the tension and in our data, these were coded as both, and hence their net effect in the overall tension pattern was quantified as zero (c) the overall contribution of each utterance was analyzed against the general emotional “climate,” but some interventions may increase the tension for some members while decreasing it for others; for instance, agreeing with one partner who disagrees with another, and (d) different kinds of utterances might have a different power to influence tension and relaxation; for instance, personal attacks might hold greater potential to increase tension than counterclaims but in our analysis, they are quantified in the same way. These methodological considerations notwithstanding, we argue that this analysis provides a way of creating a simple visualization of the overall direction of the socio-cognitive tension-relaxation patterns that might emerge in argumentative exchanges, which is useful for our research purposes. The analysis, independently conducted by the first two authors, showed an agreement of 95% and a Cohen's kappa value of 0.9.

**Table 2:** Coding categories for the analysis of the socio-cognitive tension/relaxation patterns (adapted from Andriessen et al., 2011).

**Tension–Relaxation**	**Sub-categories**	**Instances from the students' discourse**
Tension (increase)	Counterclaims	*65 Elena: But almost no one consumes a balanced omnivorous diet*
	Taking stance, persisting	*75 Elena: I would give it a 7*
	Requests for justification or clarification	*146 Santiago: Is it for the score?*
	Personal attacks, accusations	*82 Elisa: Shut your mouth, boy*
	Sarcasm, exasperation	*114 Alfonso: I give less to the vegetarian, but… but a 10 for compensating, right? No way, no way*
	Interrupting	*133 Santiago: [simultaneously] I give it a 5, I know, I know what we are talking about!*
	Showing opposition, ignoring, irrelevancy	*142 Alfonso: No, it's not like that*
	Giving directives	*147 Elena: Be rational*
Relaxation	Building	*78 Elena: [nods] And other diseases*
(decrease of tension)	Compromise, concession	*89 Elena: We have to do an average if we can't agree*
	Humor	*84 Alfonso: It is so tasty*
	Focusing, change of focus	*109 Elena: And what about the vegetarian one?*
	Clarification	*14 Elena: No, for instance, the omnivorous diet… maybe you don't cut so many trees and it's less… it leads to… is better for the environment*
	Showing agreement or approval, encouragement, confirmation	*55 Elisa: Exactly. Especially with meat*
	Following up, Giving a turn	*96 Santiago: So ten, plus four, divided into 2… equals 7*

## Results

To explore how the epistemic outcomes achieved in argumentative exchanges relate to interlocutors' epistemic aims and the socio-cognitive climate sustained, first, we examined how the epistemic status of both options changed throughout the debate. Then, we addressed the characterization of the epistemic and non-epistemic aims of the participants, and their evolution. Finally, we examined the emerging socio-cognitive tension-relaxation patterns and their relationship with the epistemic outcomes achieved.

### Evolution of the epistemic status of the options and participants' epistemic aims

**Figure 1:**
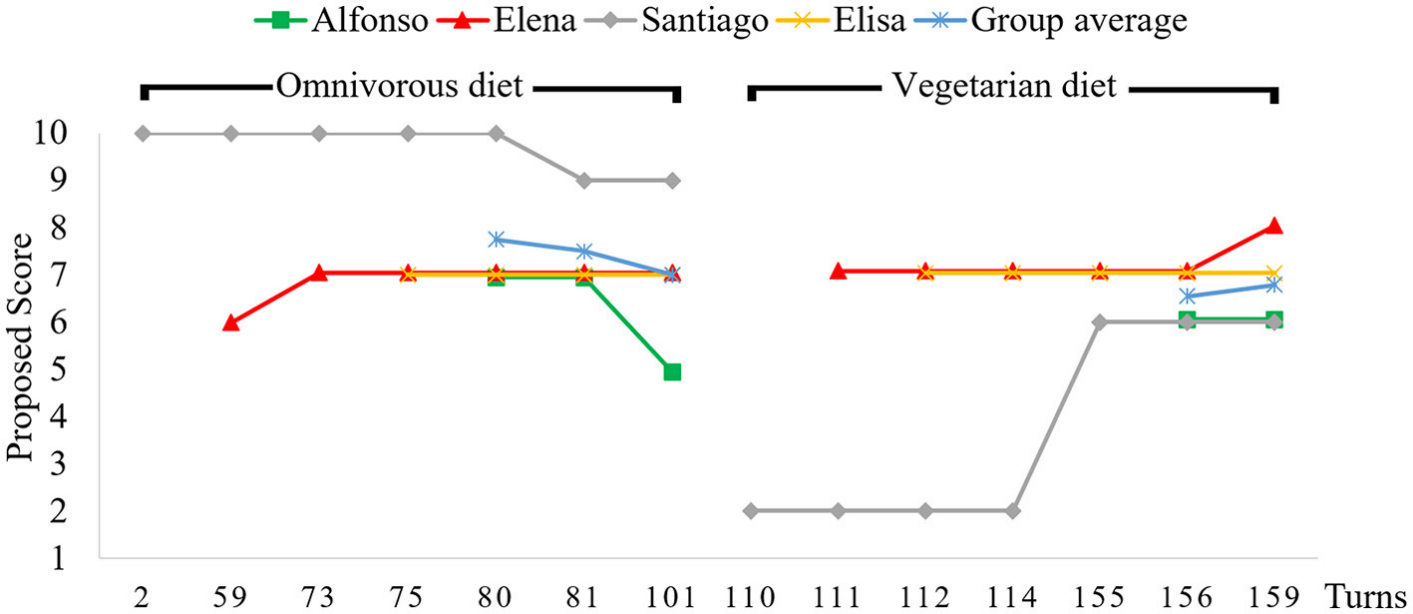
Evolution of the proposed scores for the nutritional acceptability of omnivorous and vegetarian diets throughout the debate.

We analyzed the modification of the epistemic statuses (i.e., their *acceptability*) of both diets (omnivorous vs. vegetarian) throughout the debate regarding the nutritional criterion by tracking the scores for each diet proposed by each participant, as displayed in Figure 1. The horizontal axis includes only the turns in which a new score was proposed or changed by any participant, and their representation is not linear to improve readability. The group average scores (in blue) are only represented after all group members had already proposed or agreed to a specific score.

From Figure 1, it is apparent that the student whose opinions/scorings changed the most was Santiago, as discussed below. The final group score was the same for both diets, seven. However, the process for arriving at it differs. The acceptability of the omnivorous diet was higher at the beginning of the debate for two students, Santiago and Alfonso. It did not change for Elisa and was initially lower for Elena. So the group average decreased through the debate. The opposite happened for the vegetarian diet where its acceptability was initially lower for Elena and particularly for Santiago and it did not change for Alfonso and Elisa; the group average increases slightly. This trend was particularly noticeable in Santiago's scores for both diets, which were initially outliers, but converged toward the group average as the debate moved forward.

We studied the students' aims to better understand the scores proposed and their modifications in the debate. The results of the analysis of the performances indicating epistemic or non-epistemic aims for each participant are summarized in Table 3.

**Table 3:** Participants' performances indicative of epistemic (E) aims and non-epistemic (NE) aims.

**Participant**	**Performances indicative of E aims**	**Performances indicative of NE aims**	**Uncodifiable**
Alfonso	29	4	12
Elena	21	2	8
Elisa	13	2	12
Santiago	12	17	8
Total	75	25	40

**Figure 2:**
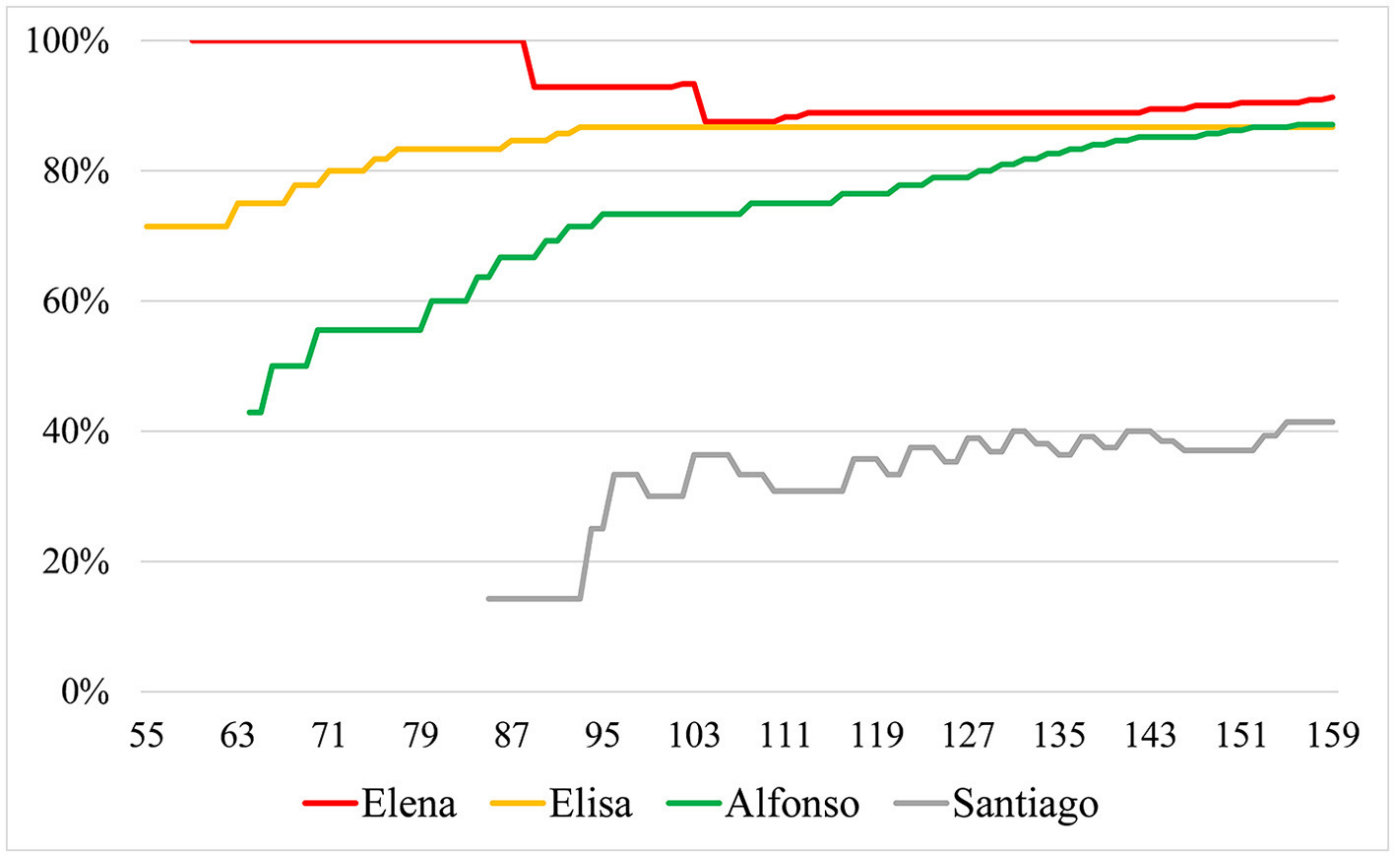
Evolution of performances indicative of epistemic aims through the debate for each participant.

Elena, Elisa, and Alfonso showed a clear predominance of epistemic over non-epistemic aims. However, Santiago's performances were indicative of non-epistemic aims and were more frequent than those indicative of epistemic ones. To better understand the characterization of the epistemic aims for each student, Figure 2 illustrates how the ratio between epistemic and non-epistemic aims changed throughout the debate. Specifically, it showed the percentage of performances indicative of epistemic aims relative to the sum of performances indicative of both aims identified for each student up to each represented turn. To exclude initial sharp fluctuations, the starting point for each student's ratio corresponded with the turn in which seven of their performances had been coded as either indicative of epistemic or non-epistemic aims.

As seen in Figure 2, most of Elena and Elisa's utterances were consistently indicative of epistemic aims, since for most of the debate they signaled a commitment to attain a better understanding of the issue and achieving scores that are properly justified. This engagement is illustrated with this fragment:

**Table:** 

	**Performances** (E: epistemic; NE: non-epistemic; U: uncodifiable)
*30 Elena:* [Reading from the handout] “*It is considered balanced the following nutritional distribution regarding the caloric content of a diet”… this is wrong*. […]	E: Detecting potential errors in the handout
*33 Elisa: No, it's right. Lipids… lipids 60, proteins 10… and fat 30*…	E: Challenging a peer's understanding
*34 Elena: It is not 50, it's 60… that's what I studied*.	E: Challenging a peer's understanding
*35 Alfonso: What difference does it make… let's move on, let's not stop at that*.	NE: Restricting the amount or quality of the evidence considered
*36 Elena:* [keeps reading the handout aloud] […]	U
*45 Alfonso: Nothing, that… read in which foods is present, to know the diets and*…	NE: Restricting the amount or quality of the evidence considered
*46 Elisa: Meh, it's all the same, but*…	NE: Restricting the amount or quality of the evidence considered
*47 Elena: But no, look, for example, mine about zinc, it says… where we can find it*	E: Providing relevant evidence
*and… ok, but, look, why is that important?* […] *So that is the problem with the vegetarian people, because… in the animals* [minerals] *are better bioavailable*. […] *That's the problem*.
*48 Elisa: I believe that… there are some* [nutrients] *that are in food from animals, where if you have to mix them to obtain them, then you have to be very careful, and*…	E: Sharing individual understandings with the group
*49 Elena: You mean like mixing lentils with rice and all that…?*	E: Asking questions to understand a peer's reasoning or to clarify the meaning of a concept

Alfonso's utterances suggest the predominance of the non-epistemic aim of finishing the task as soon as possible (35, 45), as he actively tries to stop Elena and Elisa from discussing and evaluating the available information. However, soon afterward, he also engages in an epistemic talk (analyzed below) discussing the problem definition with Elena and Elisa, agreeing with them about the assignment of a justified nutritional score of seven to the omnivorous diet. From this point on, Alfonso's interventions are consistently indicative of epistemic aims, as illustrated by the steady upwards trend in Figure 2. He adopts a central role in the debate, particularly in his interactions with Santiago, discussed below (see turns 90–97 and 116–156).

In contrast, most of Santiago's utterances can be interpreted as indicative of non-epistemic aims, particularly at the beginning of the debate, as represented in Figure 2. He interrupts the teacher's explanation, assigning an unjustified ten score to the omnivorous diet as early as turn two. He does not participate in evidence evaluation (30–50) and in turn 58 he suggests a *voting and averaging strategy*, which, as discussed later, potentially discourages epistemic talk. Later, he continues to engage in non-epistemic performances when he disregards others' arguments and does not provide justifications for his scores (*120: But I give it a 2*), or even when he provides them on his peer's insistence they are based on *non-evidence* or *pseudo evidence* (Kuhn, 1991) such as in turn 99 (*But I give it a 9. Because that… I still count it… as a 9*) or turn 129 (*Because it seems… wrong to me)*. However, Santiago's epistemic performances undergo an increase in frequency, from 14% in turn 85 to 41% in turn 159, which suggests a shift toward the adoption of epistemic aims. His later interventions in the following session when discussing other criteria support this interpretation as he then engages in epistemic performances such as asking questions to clarify the meaning of a concept (*234: Teacher, what is the gross added value?)*, providing relevant evidence (*s2-33: People working in the primary sector only cover 4% of the total population)*, or detecting errors on the informational handout (*s2-158 Santiago: Here there is an incongruity!)*.

We may summarize the evolution of the epistemic status of both options as a convergent process. The participants who showed a predominance of epistemic aims proposed similar, consistent scores. This convergence of scores was concurrent with a convergent progression in the balance between epistemic and non-epistemic aims, noticeable in the increase of epistemic aims for the participants that did not show a predominance of epistemic aims from the beginning. For instance, Alfonso, and particularly Santiago, whose proposed scores experienced the most dramatic changes throughout the debate.

### Socio-cognitive tension-relaxation patterns and epistemic outcomes

Next, we examined the patterns of socio-cognitive tension-relaxation that emerged during the debate, exploring their relationship with the epistemic outcomes achieved and with the shifts in participants' epistemic aims.

**Figure 3:**
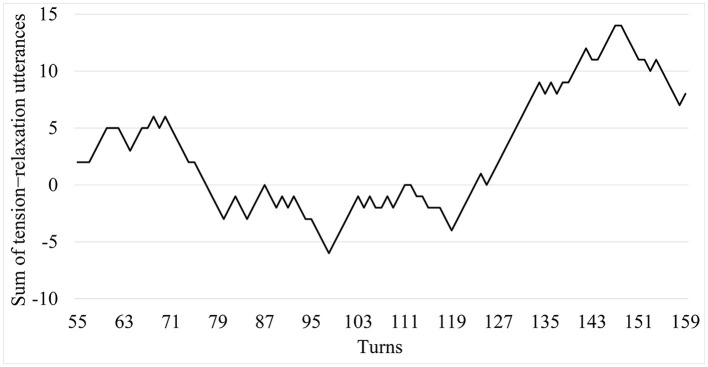
The pattern of socio-cognitive tension-relaxation during the debate.

Figure 3 displays the changes in the tension-relaxation pattern throughout the debate, quantified as the number of tensed statements minus the number of relaxed statements identified in each turn.

The first significant instance of tension increase arises in turns 58–70 during an argumentative conflict led by Elena and Alfonso:

**Table:** 

*58 Santiago: But no, look, each one of us gives a score, and then we do the average*.	NE	T
*59 Elena: So I would give it a 6, a “C”, because… lots of people that eat meat are having a lot of cardiovascular issues*.	E	T
*60 Alfonso: But we are talking about, I mean, about a balanced diet*.	E	T
*61 Elena: Sure, ok, but… uh… almost no one eats like that… almost no one consumes a balanced omnivorous diet*	E	R/T
*62 Alfonso: If you follow a balanced omnivorous diet*…	E	
*63 Elisa: It's alright*	E	R
*64 Alfonso: It's great*.	E	R
*65 Elena: But almost no one consumes a balanced omnivorous diet*.	E	T
*66 Alfonso: I know, but we have to include that case, right?*	E	T
*67 Santiago: Well, we need to think that*…	U	T/R
*68 Elisa: No, you have to include every case*…	E	T
*69 Elena: Every one*.	E	R
*70 Alfonso: In general?*	E	T
*71 Elisa: Of course*.	E	R
*72 Alfonso: Ok [nods]*	U	R

**Figure 4:**
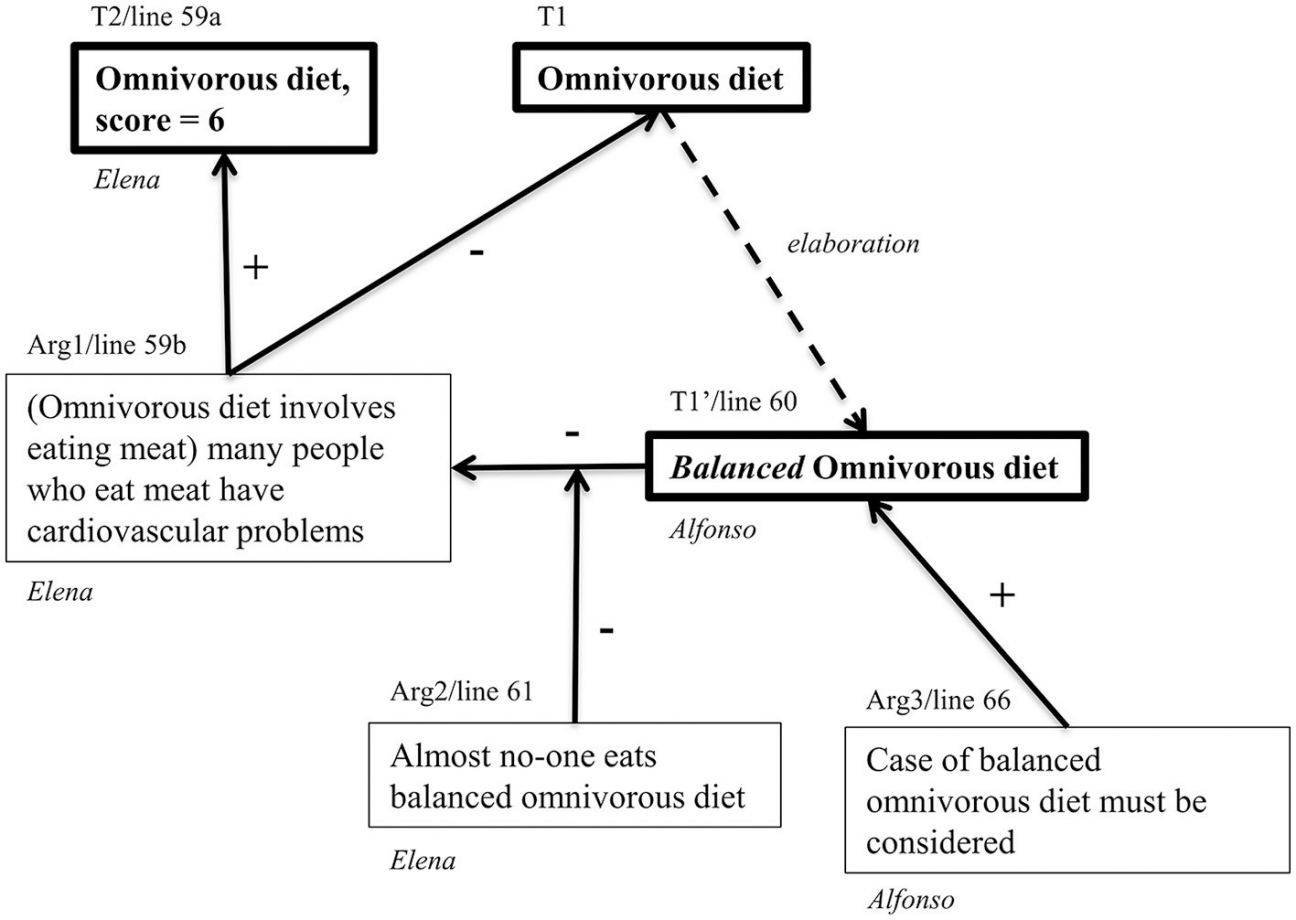
Argument diagram of turns 59–72. Boxes with thick lines are claims, that become theses (T1, T2) once argued; boxes with simple lines are (counter-) arguments; “+” and “–” are arguments for or against; dotted arrows represent elaborations of theses, noted T′.

The argumentative structure of this episode, in which there is a predominance of epistemic performances, is represented in diagrammatic form in Figure 4. The diagram represents a reconstruction (van Eemeren et al., 1993; van Rees, 2001) of the structure of the main arguments and theses expressed. As van Rees (2001) points out, reconstructing argumentation involves identifying the implicit and indirect meaning of the discourse according to the elements of a particular *model* of argumentation. In this case, the model combines elements of Toulmin (1958) structures and speech acts in argumentative discussions (van Eemeren and Grootendorst, 1984). In addition to argument structures, the diagram shows operations of *elaboration* of theses, for example, when the “omnivorous diet” option is made more precise or restricted (Naess, 1966) as a “*balanced* omnivorous diet”.

**Figure 5:**
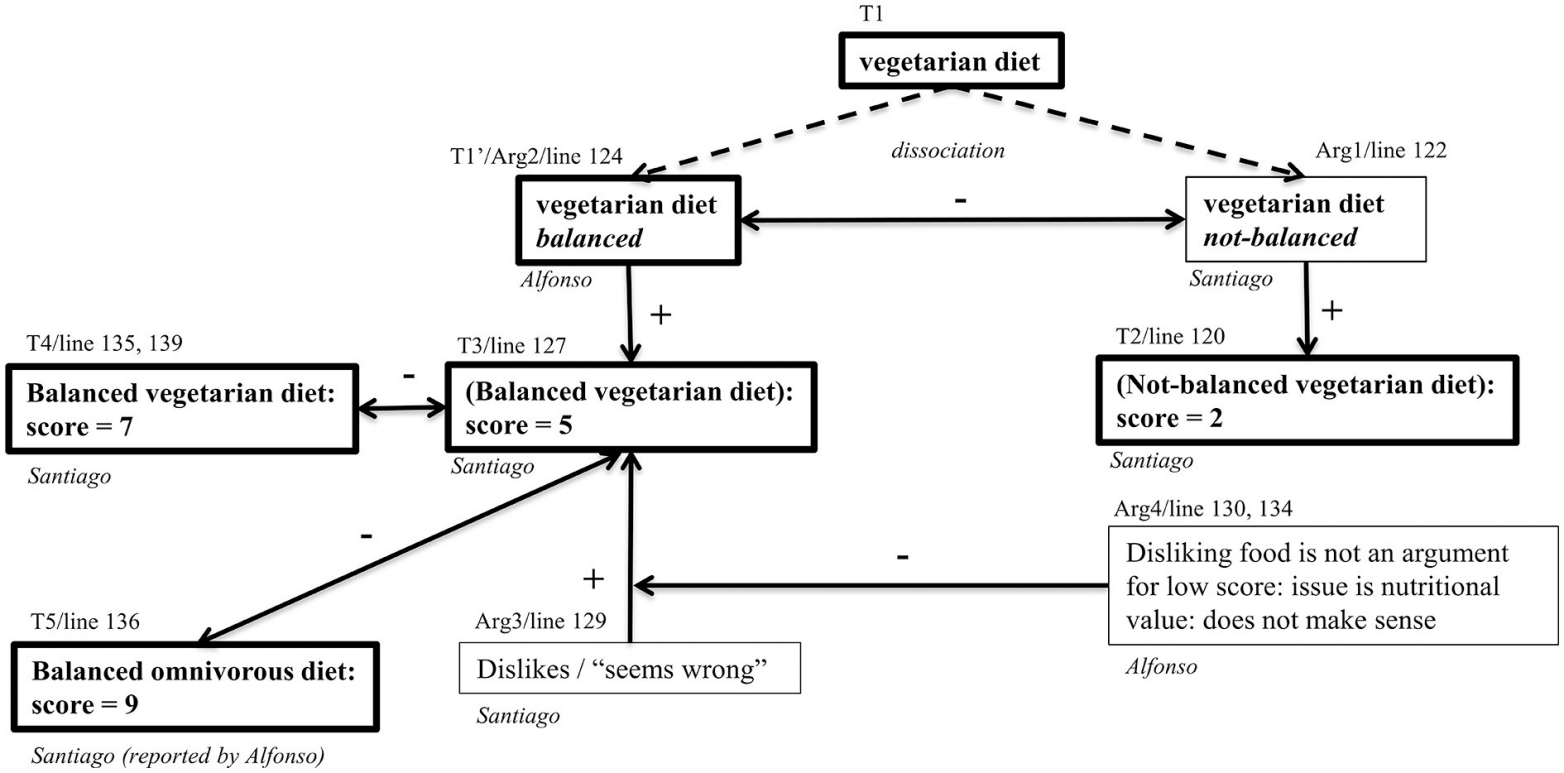
Argument diagram of the episode on scoring vegetarian diet. Boxes with thick lines are claims, that become theses (T1, T2) once argued; boxes with simple lines are (counter-) arguments; “+” and “–” are arguments for or against; dotted arrows represent elaborations of theses (rather than arguments), noted T′. The bi-directional arrow expresses that the boxes it links are mutually contradictory, each being an argument against the other.

In diagrams of the kind shown in Figure 4 (see also Figure 5), the line of the transcript that is reconstructed argumentatively is shown above the thesis boxes. For example, T1′, “*balanced* omnivorous diet” corresponds to line 60, “*But we are talking about, I mean, about a balanced diet.”* In other cases, a reconstructed argument may span several turns. Argumentative relations are rarely explicitly made by arguers and thus need to be understood in context.

The main thesis discussed (T1) is the omnivorous (O) diet. Elena claims that it should have a score of 6, not higher, providing the supporting argument (+) that it involves eating meat, and many meat-eaters have cardiovascular problems. Alfonso engages in the negotiation of the meaning: T1, O, should be understood as a *balanced* O (T1′). This constitutes his argument against Arg1: an O diet does not necessarily cause cardiovascular problems if it is balanced. Elena (61) criticizes the argumentative *relation* rather than T1′ argument itself: O diet is not generally balanced. Alfonso (Arg3, 66) replies that the balanced O diet must nevertheless be taken into consideration. Elisa and Elena accept contemplating this case, but not exclusively: it must be considered in the context of how the O diet is generally carried out, which is not in a balanced way, to which Alfonso finally agrees. Conflict is solved and the students align their epistemic statuses: Elena has succeeded in defending her claim of the score of six for the O diet and in countering Alfonso's counterarguments, but there is some co-construction as well because they agree in considering the balanced O diet or, in Elisa's words, that they have to “include every case”.

In this exchange we observe several epistemic outcomes: arguments and counterarguments constructed, links established and questioned, elaboration of theses, and dissociation of O diets into balanced and not balanced. Simultaneously they are regulating their engagement in argumentation: they discuss which form of the options should be evaluated, whether an ideal balanced O diet or one that would be representative of how this diet is currently being followed by the general population. This negotiation of the problem definition has implications for how they should tackle the task, scope, and strategy for their evaluation, which is further operationalized later (92–96 and 148–150). This episode in which conflict is solved and epistemic outcomes are co-constructed seems to be a turning point for Alfonso, who up to this point showed a predominance of non-epistemic aims, which are not identified thereafter (see Figure 2).

Tension decreases shortly after an agreement is reached among Alfonso, Elena, and Elisa. They concur in scoring the O diet with a seven, collectively co-constructing a justification for it (73–80, omitted). However, Santiago does not participate nor criticize it, proposing instead an independent score (81), enacting the *voting and averaging* strategy that he had previously proposed (58). In this context, we interpret the proposal for this strategy as a way of preemptively avoiding engagement in epistemic talk, preventing the modification of the epistemic status of the options. After Santiago's lack of participation in evidence evaluation (30–50) and in the co-constructed justifications for a seven score, his unjustified nine score for the O diet is heavily rejected, especially by Elisa: tension rises until turn 87.

**Table:** 

*81 Santiago: I'm giving it a 9*.	NE	T
*82 Elisa: Shut your mouth, boy*.	U	T
*83 Santiago: For me… I think the omnivorous diet… it is ideal. […]*	NE	R
*88 Teacher: Well, if you cannot agree on a mark, so use an average*.	U	R
*89 Elena: We have to do an average if we can't agree*.	NE	R
*90 Alfonso: Ok, look, I'm explaining to you, the omnivorous diet is very good, I already said so, if it's balanced, if it's balanced is a 10, you get everything, you eat everything, it's awesome*.	E	T
*91 Elisa: It is perfect*.	E	R
*92 Alfonso: But it's not always balanced, so you have to include every case, what it deserves. And in every case, if we eat a lot of meat, you are getting*…	E	T
*93 Elisa: Obesity*.	E	R
*94 Santiago: But then it's not a 7, it's a 5 or a 4*.	E	R
*95 Alfonso: So that's what I'm saying, that we have to put ourselves… in the average*.	E	R/T
*96 Santiago: So 10, plus 4, divided by 2… equals 7*.	E	R
*97 Alfonso: Alright! Seven! There you go*.	U	R
*98 Elisa: [Applauds]*.	U	R

Possibly induced by the perception of excessive socio-cognitive tension at this point, and perhaps also in the interest of time, the teacher accepts the validity of the averaging strategy, suggesting its implementation in turn 88, which could relax the tension, but also restrict potential epistemic outcomes developed in a further attempt to reach consensus. Santiago's proposal for this strategy had remained unaddressed, but after the teacher's recommendation, Elena accepts its adoption (89). However, Alfonso and Elisa do not follow up by casting their votes as they still apparently aim to solve the disagreement and reach a consensus. Through dialogue (90–93) they reconstruct their argument, explicitly considering what they had agreed upon, which was that they should consider balanced and “unbalanced” O diets. This agreement is seemingly appropriated by Santiago (94–96), in interaction with Alfonso. Specifically, they operationalized the problem by separately considering both extreme cases (idealized-balanced and realistic-unbalanced diets) and estimating an average score. Santiago follows Alfonso's argument, reaching the same conclusion as they did with a score of seven for the O diet. Alfonso and Elisa (97–98) give him encouraging feedback. The socio-cognitive climate has relaxed from turn 88 to 98. Agreement (and thus, alignment of epistemic statuses) is reached, without resorting to the voting strategy.

Immediately thereafter, Santiago retracts his score, advocating a nine with no valid justification, resulting in tensions flaring up again.

**Table:** 

*99 Santiago: But I give it a 9. Because that… I still count it… as a 9*.	NE	T
*100 Elisa: Fuck, Santiago*.	U	T
*101 Alfonso: Ok, I give it a 5, and there we go [laughs]*.	U	T
*102 Elena: Only because it's tasty?*	E	T
*103 Santiago: What are you saying, dude? No… a 9 because… it's true. It gives you everything*.	E	T
*104 Elena: We have to do the average among us, seven… three sevens and one nine*…	NE	R
*105 Alfonso: No, no. You and she gave it a 7, he, a 9, and me, a 5. It equals 7. There we go*.	U	T
*106 Elena: Ok [laughs]. So that's it*.	U	R
*107 Santiago: Put a 7!*	NE	T/R
*108 Alfonso: It is indeed a 7*.	E	T

Santiago's discursive move is strongly rejected by Elisa. Alfonso seemingly desists from addressing Santiago's unjustified position and *artificially* changes his score to render Santiago's without effect by way of dismissing him and raising the tension. Our interpretation is that Santiago is implicitly being accused of violating the rules of debate in two senses: lacking argumentative coherence, and hindering progress, repeating an argumentative move with no further justification. Elena (102), arguably driven by epistemic aims, persists in her effort to uncover the reasoning behind Santiago's position, pointing to gastronomic preferences. Her discursive move may be interpreted as yet another accusation of violating a rule, namely overstepping the scope of the debate, which should be restricted to the nutritional criterion. Santiago rejects her accusation and Elena desists, moving the debate forward by implementing the averaging strategy. She then reiterates all the proposed scores, not taking Alfonso's (101) tweaked score seriously. Alfonso, however, stands by it, which is then accepted by Elena. Santiago, perhaps due to excessive pressure, exasperatedly concedes (107), but it is a forced, disinterested concession, with no real agreement: they change the focus, moving forward. The tension is sustained and the disagreement is not solved nor carried further until its ultimate consequences. At this point, the participants have tried but failed to align their epistemic status and agree on the regulation of the rules of debate.

A similar event takes place immediately thereafter when they evaluate the vegetarian (V) diet, and Santiago proposes a low score with no justification:

**Table:** 

*109 Elena: And what about the vegetarian one?*	U	R
*110 Santiago: A 2 [the other group members laugh, Santiago shrugs]*	NE	T
*114 Alfonso: [laughing] I give it less to the vegetarian one, but… a 10 to compensate, right? No way, no way. I give it…. come on, man, speak right, I'm speaking right, dude*.	U	T/R
*115 Santiago: Ok, ok*.	U	R
*116 Alfonso: […] don't you give it a 2… an organized vegetarian diet can cover*…	E	T/R
*117 Santiago: Also the omnivorous one*.	E	T/R
*118 Alfonso: That's it!*	U	R
*119 Elena and Elisa: Ah! [They pound the table]*	U	R

The group rejects Santiago's unjustified score, by displaying emotions (sarcastic laughter), thereby increasing the tension. Alfonso then acknowledges that he does not consider the V diet as nutritionally adequate as the O one, and yet, he shows a disposition to fake his vote again to balance what might be considered an unreasonably low score. In other words, he uses humor about “tweaking” the score for increasing the pressure on Santiago, which continues from their previous exchange. Alfonso also exhorts Santiago to “speak right” as he is doing, interpreted as an encouragement to follow the rules of debate, which is, this time, seemingly accepted by Santiago (115), de-escalating the tension. Then, Alfonso points out that a V diet could cover all nutritional needs, which is implicitly accepted by Santiago (117). The rest of the group reacts by displaying enthusiastic emotions, relaxing the tension, as they presumably consider that, once Santiago acknowledged Alfonso's statement, he would accordingly modify his score to avoid a lack of coherence. However, Santiago insists on his unjustified score:

**Table:** 

*120 Santiago: But I give it a 2*.	NE	T
*121 Alfonso: Why?*	E	T
*122 Santiago: Because, because it doesn't seem… balanced to me*.	E	T
*123 Elisa: Really, hu?*	U	T
*124 Alfonso: But it can be balanced sometimes, dude!*	E	T
*[…]*		
*127 Santiago: If the thing is balanced… I give it a 5*.	E	T
*128 Alfonso: Because you don't like it*.	E	T
*129 Santiago: Because it seems… wrong to me*.	NE	T
*130 Alfonso: But we are not talking about the food, we are talking… [about nutrients]*	E	T
*133 Santiago: I give it a 5, I know, I know what we are talking about!*	NE	T

Tension progressively increases during turns 120–134, as disagreement is not solved. Alfonso demands a justification for Santiago's score, and upon getting an unsatisfactory answer, prompts additional exclamations from Elisa. This can be interpreted as an implicit denouncing of Santiago's failure to comply with the rules for coherence and for a valid defense of a standpoint when challenged. Alfonso suggests, as Elena did in turn 102, that Santiago might be influenced by preferences beyond the space of debate on nutrition, which is again denied by Santiago.

**Table:** 

*134 Alfonso: But what you are saying doesn't make sense*.	E	T
*135 Santiago: Ok… so put a 7*.	NE	R
*136 Alfonso: But listen, you are saying that the omnivorous diet is a 9, because it has all the nutrients, but a vegetable diet that has all the nutrients is a 5 because it's vegetable*.	E	T
*137 Santiago: It's a 5 because it's vegetable, for sure*.	E	R
*138 Alfonso: But that doesn't make sense! [Elisa and Elena laugh]*	E	T
*139 Santiago: Ok, so then give it a 7!*	NE	T/R
*140 Alfonso: No. We have to engage in argumentation, it's not that way*…	E	T
*145 Alfonso: But no… God! Come on, listen*.	U	T
*146 Santiago: Is it for the score?*	NE	T
*147 Elena: Be rational*.	E	T

Alfonso (134, 138) keeps pointing out Santiago's lack of coherence in his evaluation of balanced O and V diets, reconstructing (136) his implicit argument to point out a contradiction: if the criterion for nutritional adequacy is the capacity to supply all necessary nutrients, and if they are comparing “balanced” versions of both O and V diets, which provide all nutrients, it is not legitimate to give them different scores. This critique is supported by Elisa and Elena's laughter and answered by Santiago's *disinterested concession* (139) for the sake of finishing the discussion as a mean to reduce tension.

In this sequence (120–147) we observe a sustained increase of tension, which arguably reaches its highest peak, followed by Santiago's attempt to relax it by means of a disinterested concession, as he did earlier when discussing the O diet. Interestingly, this time, Alfonso explicitly rejects it, stating that “we have to engage in argumentation,” and alongside Elena, keeps exerting pressure and encouraging Santiago to “be rational,” to follow the rules of the debate, and thus to adopt a sounder epistemic stance. This might also be interpreted as a manifestation of the belief that a decision achieved through disinterested concessions would not be reliable. At this point we identify Alfonso's clearest declaration of epistemic aims: they have to argue properly even if that implies a high level of socio-cognitive tension; in other words, the epistemic dimension must take precedence. Santiago even inquires on the motives for sustaining such tension: “is it for the score?” (146), implicitly suggesting that, if what is really at stake is a task-oriented goal (i.e., the score), he is ready to concede to prioritize the socio-relational dimension. However, Alfonso's refusal to accept such disinterested concession and his disposition to keep the tension (140, 145) suggests that he is not driven by task-oriented goals, but by knowledge-oriented, epistemic ones: they must reach a collective agreement, for the right reasons, and according to the proper rules of debate.

This episode is summarized in an argument diagram (Figure 5) that highlights the argumentative and conceptual operations involved (i.e., the epistemic outcomes).

Figure 5 illustrates two main characteristics of the argumentative sequence on the acceptability of the vegetarian diet. Firstly, the sequence turns on a classical move in argumentation, that of “dissociation” (Perelman and Olbrechts-Tyteca, 1958; Baker, 2002); in this case, the concept of “vegetarian diet” is dissociated into two possible sub-concepts, “balanced” and “unbalanced.” This distinction is introduced by Santiago to support his low score of two, for the purportedly unbalanced V diet. The other students maintain the existence of the opposite, a balanced version of the diet. The second characteristic is the diversity of ways in which Santiago's claims are shown as invalid: (i) his ‘argument' against any form of V diet in terms of his simple dislike for it is claimed by Alfonso to be an unacceptable argument, and irrelevant given that the issue here is nutritional value; (ii) Alfonso shows that Santiago's views are internally contradictory since for “balanced” diets, O or V, he gives very different scores (9 and 5, respectively); (iii) Santiago's quick change of score for the balanced vegetarian diet from 5 to 7 when faced with criticism of his view is not motivated in epistemic terms and contradicts himself but (as the diagram shows) his new score has no argument in its favor.

At the end of this episode, Alfonso, in discussion with Elena, reconstructs their argument once again, the validity of which is finally accepted by Santiago (153):

**Table:** 

*148 Alfonso: Listen, look. We are assessing… the capacity of the diet for getting all the necessary nutrients, so, for the omnivorous diet, it is easy to reach the nutrients, but it is also easy to go too far, so then you need to do the average*.	E	T/R
*149 Santiago: An average that is 7, yes*.	U	R
*150 Alfonso: Ok, so in the vegetarian diet you have to do the same, assessing… it's difficult to end up having all the necessary nutrients with a vegetarian diet, you have to balance it very well*.	E	R
*151 Elena: But it's not impossible*.	E	R
*152 Alfonso: But you can do it. You have to take that into account*.	E	R/T
*153 Santiago: Ok. So here I gave it*…	E	R
*154 Alfonso: It's not a 2, dude*.	U	T
*155 Santiago: Then I give a 6 for the vegetarian*…	E	R
*156 Alfonso: Ok, that makes sense. Ok, I give it a 6 also*.	E	R

Santiago accepts it this time, changes his score accordingly, and proposes a 6, which converges toward the group average. This move is acknowledged by Alfonso, who legitimizes Santiago's shift, providing positive feedback (156). Thus, after a prolonged sequence of high tension, the socio-cognitive climate has finally relaxed, even before the turn in which Santiago finally concurs with the rest of the group. He revises his position and consequently rectifies his scores. Epistemic statuses are seemingly aligned, and agreement is finally reached.

## Conclusions

This paper analyzes the interplay between epistemic and socio-cognitive dimensions in argumentation in a case study. While not generalizable, we believe that our findings can shed some light on certain critical aspects of the interconnections among epistemic aims, the patterns of socio-relational climate sustained, and the epistemic outcomes achieved in argumentative interactions.

The results indicate that epistemic aims predominate for three of the four students, which suggests that, overall, the task design and implementation were successful in enabling their epistemic aims and performances. The scores proposed by them were similar and largely consistent with the literature about the nutritional adequacy of V and O diets (Leitzmann, 2014; Sabaté and Soret, 2014), which differs from public consideration of O diets being nutritionally better than V ones (de Bakker and Dagevos, 2012; Pohjolainen et al., 2015). The contributions of the fourth member, however, are mainly indicative of non-epistemic aims for the greater part of the debate, corresponding with the proposal of scores that are outliers in comparison to those of other members. Thus, there seems to be a correspondence between the epistemic status of the options and the epistemic aims adopted by the participants. The balance between epistemic and non-epistemic aims is dynamic, involving changes throughout the debate. In our data, we observed a convergence of both participants' epistemic aims and of the epistemic statuses of the options; a gradual adoption of epistemic aims coincides with scores converging toward the group average.

The analysis of the socio-cognitive patterns of tension-relaxation might help us understand how these convergent processes and epistemic outcomes were developed. Most of the epistemic outcomes identified were produced in sequences with the following socio-cognitive pattern: tension arises, and relaxation follows. This suggests that the group was successful in combining critical and co-constructive discursive moves (Asterhan, 2013) in a fluctuating socio-cognitive climate, and supports the idea that tuning at the cognitive level is related to tuning at the socio-cognitive level (Andriessen et al., 2011).

Our findings suggest that these tuning processes are yet related to another one: the tuning of participants' aims. The changes in epistemic aims are noticeable for two participants: Alfonso and Santiago, but there are differences in how they change and their relation to the socio-cognitive climate. Alfonso showed some instances of non-epistemic aims at the beginning of the debate, but after the first socio-cognitive conflict, which was successfully resolved (and the climate, thus, relaxed), his performances were consistently indicative of epistemic aims: the change is drastic. This suggests that, rather than being necessarily in conflict with epistemic matters, the socio-relational dimension, when it is successfully dealt with, could be related to the promotion of epistemic aims. It must be noted that in the case of Alfonso, part of his initial position is integrated into the group's agreement, which might have facilitated his transition toward epistemic aims.

The case of Santiago is different: his discrepancies with the rest of the group are much more pervasive, becoming a source of conflict and tension throughout the debate. His position is not integrated into the group agreement as the others do not accept the validity of his arguments. Despite prolonged increases in socio-cognitive tension, in the end, the group manages to reach an agreement. Santiago concurs with his partners and shows a gradual increase of epistemic aims throughout the debate. Our interpretation is that these changes in epistemic status and epistemic aims might have happened not despite the high tension sustained, but rather because of it. Had the students prioritized a favorable socio-emotional climate, particularly after a reasonable exploration of their irreconcilable differences, the final agreement would have not presumably been reached, implicit premises might have remained misaligned, and Santiago's position would have not shifted. That is what seems to have happened at the end of the discussion about the omnivorous diet as they give up their efforts to explore a mutual understanding and choose to dismiss Santiago's position. At that point they are not fully prioritizing epistemic aims: they choose to decrease the tension by moving forward, even if the conflict remains unresolved. But later, when a similar situation unfolds discussing the vegetarian diet, they instead keep their epistemic aims until their ultimate consequences, deeply pushing the levels of tension. They then prioritize the epistemic dimension over the socio-relational one, and, in doing so, they reach a mutual agreement.

The rejection of the validity of Santiago's arguments, and thereby the refusal to integrate them into the group consensus, might be explained by the consideration that, in the eyes of the other members, his discursive moves violate the norms for reliably engaging in argumentation and decision-making. The others explicitly point out these norms and criteria, commending him to properly follow them. Thus, there seems to be yet another process involved where the tuning of the enacted epistemic processes and the ideals and conditions should be met to reliably produce epistemic outcomes. Our findings suggest that, when driven by epistemic aims, participants engaging in argumentation can refine the conditions by which they carry out this practice. They also suggest a development in the participants' epistemic understanding of the norms and rules governing the argumentative discourse, in alignment with Kuhn et al. (2013) findings. In the literature, the beliefs about the conditions to be met to reliably perform processes such as argumentation are considered generally tacit (Baker, 2009; Chinn et al., 2011), but in our study, we identified several instances of negotiation of these conditions in contexts of increasing socio-cognitive tension, such as requirements for argumentative coherence, beliefs about the reliability of voting vs. consensus-seeking, or the invalidity of disinterested concessions.

Overall, our findings highlight some of the complex relationships that may stem from the interplay among the participants' aims, the socio-cognitive climate, and the epistemic outcomes achieved. In light of our results, we argue that particular epistemic and non-epistemic aims (and the balance between them) that were adopted by each participant are likely to evolve because of the socio-cognitive climate and the influence of the group epistemic outcomes achieved, particularly the arguments built and their persuasiveness. This, in turn, potentially affects the epistemic processes enacted by each participant, which influence how the rest of the debate is carried out, in an iterative, back-and-forth manner, in which participants can engage in the regulation of the norms and criteria to enact epistemic processes and further adopt epistemic aims. In the group analyzed, this dynamic results in an increasingly convergent trend of shared epistemic aims and processes, which appears to be related to the convergence of the proposed scores (i.e., epistemic statuses). Overcoming conflict seems to encourage the adoption of epistemic aims, as illustrated in the case of Alfonso.

The value of this case study, we believe, emanates not only from the illustration of this complex interplay but from the fact that, in this particular case, the students attained sophisticated epistemic outcomes, including self-regulation, while sustaining a high degree of socio-cognitive tension. When faced with the challenge of balancing the epistemic and socio-relational dimensions, they *prioritize the epistemic dimension*. Our findings potentially challenge the consideration of socio-relational concerns as rather an obstacle to knowledge construction. In this regard, authors such as Thiebach et al. (2016) have pointed out that socio-cognitive conflict often needs to be further stimulated. Stewart and D'Mello (2018) have found negative correlations between positive perceptions of groups' agreeableness and their learning outcomes, suggesting that, prioritizing agreeability, and minimizing conflict, participants might promote favorable subjective outcomes at the expense of learning. Our findings align with theirs.

Thus, the results of this study call for further investigation on what constitutes an appropriate or productive level of interpersonal tension for promoting epistemic aims and outcomes, and upon which factors it may depend. Is it context-specific? To what degree does it depend on the personal traits of the participants, such as their character or their cultural and personal identities? How does the regulation of socio-cognitive tension relate to the perceived right to speak and participate in a conversation (Clarke, 2015)? What role do social relationships within the group, their closeness, and their friendship, play? Is this process dependent on their individual or group interest in the topic, or their previous knowledge about it? Can the capacity to tolerate socio-cognitive tension be enhanced? By which instructional approaches? Is it related to metacognitive or metamotivational experiences (Efklides, 2011; Miele and Scholer, 2018)? These are questions that we believe may be worth exploring in future research.

Another interesting research direction is deepening our knowledge about beliefs and regulations on how to reliably engage in epistemic processes such as argumentation and decision-making. Duncan and Chinn (2016) argue that instructional interventions should consider the role of norms and epistemic criteria in argumentation, advocating for further elucidation about how such norms are engendered and for determining their impact on argumentation competency. Our findings suggest that socio-cognitive tension might play an important role in the regulation of such norms and rules. Considering these results, we propose the development of further research and educational initiatives directed at enhancing students' ability to establish, defend, negotiate, and refine the conditions under which argumentation and other group procedures should be enacted. In this sense, it might be worth exploring how different types of dialogue, involving different criteria of retractability or agreement between distinct positions, are likely to be governed by certain rules of the dialogue which affect the application of socio-cognitive tension, development of epistemic criteria, and reaching more specific or unspecific agreements.

Our study presents some limitations, many of which originate from the study design. There are some analytical limitations, addressed in the Methods section, such as the lack of consideration of the relative potential of some utterances to increase or decrease socio-cognitive tension, the high level of interpretation necessary for the analysis of epistemic aims, and the non-exhaustivity of the coding scheme, which could be further developed through the incorporation of additional categories stemming from the analysis of additional data in a wider range of argumentative contexts. As a case study, our findings are not generalizable. Following the methodological approaches advocated in the literature for exploring EC in social settings (Chinn and Rinehart, 2016; Clément, 2016; Greene et al., 2016a), we have developed a fine-grained analysis at the micro-level, studying cognition-in-practice (rather than declarative knowledge) in a specific context. Therefore, our approach necessarily limits the applicability of our findings. Our intent is not to portray what generally happens in classrooms but to study a case in which sophisticated epistemic outcomes are produced and epistemic aims are adopted and try to understand which conditions allowed for them and how they could be encouraged. We conducted this study and selected this particular group and discussion not because we believe it is representative, but because it meets certain criteria, which potentially allows for a better understanding of the complexities of the interplay between the epistemic and socio-cognitive dimensions, which might be of research and instructional relevance. Future studies are likely to be enriched by a better account of the social relationships among the participants and their personal backgrounds. The analysis of the argumentative interactions could be further explored through the incorporation of non-verbal and multimodal approaches (Heller, 2021). The educational implications of this study are related to the admission or even encouragement of a certain degree of productive socio-cognitive tension and conflict through instructional strategies and prompts, and to the design of argumentative learning environments, which, through the incorporation of further research, could promote a broader disposition to adopt epistemic aims and refine epistemic processes, including the encouragement of socio-cognitive climates and peer-to-peer interactions leading to them.

## Data availability statement

The raw data supporting the conclusions of this article will be made available by the authors, without undue reservation.

## Ethics statement

Ethical review and approval was not required for the study on human participants in accordance with the local legislation and institutional requirements. Written informed consent to participate in this study was provided by the participants' or their legal guardian/next of kin.

## Author contributions

All authors listed have made a substantial, direct, and intellectual contribution to the work and approved it for publication.

## Funding

This research work was supported by the Spanish Ministry of Science, Innovation, and Universities, and partly funded by the European Regional Development Fund (ERDF) (Grant Code: PGC2018-096581-B-C22).

## Conflict of interest

The authors declare that the research was conducted in the absence of any commercial or financial relationships that could be construed as a potential conflict of interest.

## Publisher's note

All claims expressed in this article are solely those of the authors and do not necessarily represent those of their affiliated organizations, or those of the publisher, the editors and the reviewers. Any product that may be evaluated in this article, or claim that may be made by its manufacturer, is not guaranteed or endorsed by the publisher.
